# A systematic review and dose–response meta-analysis of prospective cohort studies on coffee consumption and risk of lung cancer

**DOI:** 10.1038/s41598-024-62619-6

**Published:** 2024-07-01

**Authors:** Maedeh Jabbari, Asma Salari-Moghaddam, Amir Bagheri, Bagher Larijani, Ahmad Esmaillzadeh

**Affiliations:** 1 Department of Community Nutrition, School of Nutritional Sciences and Dietetics, Tehran University of Medical Sciences, P.O. Box 14155-6117, Tehran, Iran; 2 Department of Biochemistry, School of Medicine, Ilam University of Medical Sciences, Ilam, Iran; 3 School of Nutrition Sciences and Food Technology, Kermanshah University of Medical Sciences, Kermanshah, Iran; 4 Endocrinology and Metabolism Research Center, Endocrinology and Metabolism Clinical Sciences Institute, Tehran University of Medical Sciences, Tehran, Iran; 5 Obesity and Eating Habits Research Center, Endocrinology and Metabolism Molecular-Cellular Sciences Institute, Tehran University of Medical Sciences, Tehran, Iran; 6 Department of Community Nutrition, School of Nutrition and Food Science, Isfahan University of Medical Sciences, Isfahan, Iran

**Keywords:** Coffee, Lung cancer, Meta-analysis, Risk factors, Cancer, Lung cancer

## Abstract

Studies on the association between coffee consumption and risk of lung cancer have been conflicting. The aim of this study was to systematically review the current evidence on the association between coffee consumption and risk of lung cancer and to quantify this association by performing a meta-analysis. A comprehensive systematic search was performed on online databases up to July 2023 investigating the association between coffee consumption and risk of lung cancer. All prospective cohort studies reporting odds ratios (ORs), rate or risk ratios (RRs), or hazard ratios (HRs) and 95% confidence intervals (CIs) in this context were included. The overall effect size was calculated using the random-effects model and statistical between-studies heterogeneity was examined using Cochrane’s *Q* test and I^2^. A total of 14 prospective cohort studies were included in this systematic review and meta-analysis. We found a significant positive association between coffee consumption and risk of lung cancer (RR: 1.28; 95% CI: 1.12, 1.47). This association remained significant when we included a pooled analysis paper and excluded 5 cohort studies (RR: 1.37; 95% CI: 1.12, 1.66). We observed no proof of significant publication bias using Egger’s test (P = 0.58). Moreover, dose–response analysis showed that each one cup/day increase in coffee consumption was related with a 6% higher lung cancer risk (RR: 1.06; 95% CI: 1.03, 1.09). In conclusion, we found a significant positive association between coffee consumption and risk of lung cancer.

## Introduction

Lung cancer is the second commonly diagnosed cancer and one of the leading causes of cancer mortality by its high fatality rate^1^. Smoking is a well-known modifiable risk factor for lung cancer followed by carcinogen exposures such as asbestos, heavy metals, and polycyclic aromatic hydrocarbons and etc^2–4^. Dietary intakes have also been shown to contribute to this type of cancer. Earlier studies suggested an inverse association between healthy dietary pattern and risk of this cancer, while high consumption of red and processed meat and total and saturated fats have been associated with elevated risk^5–7^.

Coffee is one of the most widely consumed beverages throughout the world next to water and tea. It contains mixtures of biochemically active ingredients such as antimutagenic and antioxidant or cancer-promoting agents including caffeine, acrylamide, melanoidins, chlorogenic acid, diterpenes, and trigonelline, which might be important in cancer development or prevention^8,9^. Previous investigations have indicated that coffee may have a protective role in type 2 diabetes, stroke, dementia, and cardiovascular diseases; however, data about cancer is conflicting^10,11^. While coffee drinking was associated with a lower risk of liver, oral, endometrial, and esophageal cancers, it was associated with an elevated risk of bladder cancer and leukemia^12^. The role of coffee intake in lung cancer has also been extensively examined, but the findings were conflicting.

Findings from a meta-analysis published in 2010 revealed a significant positive association between coffee consumption and risk of lung cancer in cohort studies. However, only five cohort studies were included in that meta-analysis^13^. Another meta-analysis on coffee consumption and risk of lung cancer, published in 2016, reached no significant association among non-smokers^14^. However, that meta-analysis included 8 cohort studies and two cohort studies were missed^15,16^. In addition, they combined results from case–control and prospective cohort studies which is not a correct method. After release of the latest meta-analysis in 2016, data from 6 large prospective cohort studies appeared^17–22^. Alternatively, some methodological concerns in earlier meta-analyses might limit their interpretation. For instance, both previous meta-analyses have included the study by Khan et al.^23^ in their analysis, while Khan et al. investigated the association between coffee consumption and risk of mortality from lung cancer, not lung cancer incidence per se. This also applies to Chow et al. study^24^. Therefore, we aimed to perform an updated comprehensive meta-analysis by including recently published studies. The aim of the present study, therefore, was to systematically review the current evidence on the association of coffee consumption and risk of lung cancer.

## Methods and materials

### Search strategy

Online databases including PubMed/Medline, Scopus, and ISI Web of Science were systematically searched up to July 2023 using following keywords: (coffee OR caffeine OR drink OR beverage OR “caffeinated beverages” OR “coffee consumption” OR “coffee intake” OR “coffee drinking” OR “caffeine consumption”) AND (“pulmonary neoplasm” OR “lung neoplasm” OR “pulmonary cancer” OR “lung cancer” OR “pulmonary tumor” OR “lung tumor”). No time of publication limitation was taken into account. However, only studies in English were included in the current study. We also performed a manual search of related articles’ references list to avoid missing any relevant published papers. Two reviewers (MJ and ASM) independently screened the output of the search to identify potentially eligible studies. Any disagreements between the two reviewers were solved by consultation with the principal investigator (AE). In addition, no attempt was made to include unreported studies.

### Study selection

Articles’ title and abstract were reviewed to find relevant publications by two independent reviewers (MJ and ASM). Publications were fully reviewed if the abstract stated that coffee consumption had been examined in relation to risk of lung cancer. Studies were eligible for inclusion in the current systematic review and meta-analysis if they met the following criteria: (1) all prospective cohort studies performed on adults ≥ 18 years of age; (2) considered coffee as the exposure variable and lung cancer as the main outcome variable or as one of the outcomes; and (3) publications in which odds ratios (ORs), rate or risk ratios (RRs), or hazard ratios (HRs) were reported as effect size.

### Data extraction

Two reviewers (MJ and ASM) independently extracted the following data from eligible studies: first author’s last name, year of publication, cohort name/country, mean age or age range (years), sex, number of subjects, number of cases, follow up duration (years), exposure assessment, outcome assessment, comparison, fully adjusted effect size (ORs, RRs, or HRs) with the corresponding 95% CIs, adjustments, and study quality score. Characteristics of included studies in this systematic review and dose–response meta-analysis are provided in Table 1.

**Table 1:** Characteristics of included studies in the systematic review on coffee intake and risk of lung cancer.

First author, year	Cohort name/country	Mean age or age range, year	Sex	No. of subjects (no. of cases)	Follow up, year	Exposure	Exposure assessment	Outcome	Outcome assessment	Comparison	OR, RR, or HR (95% CI)	Adjustments	Quality score
Kudwongsa et al., 2020	Khon Kaen Cohort Study (KKCS)/Thailand	51	M/F	12,668 (138)	15	Coffee	Structured questionnaire	Lung cancer	Registries	Yes vs. no	0.54 (0.35, 0.84)	1, 2, 3	6
Schmit et al., 2020	Southern Community Cohort Study (SCCS)/USA	Cases: 55.3 Controls: 55.6	M/F	Cases: 511 Controls: 3285	–	Coffee	FFQ	Lung and bronchus cancer	Registries	≥ 2 times/d vs. < 1 time/d	1.06 (0.77, 1.45)	1, 2, 3, 4, 5, 6	6
Seow et al., 2020	Singapore Chinese Health Study (SCHS)/Singapore	45–74	M/F	61,321 (1486)	17.7	Coffee	FFQ	Lung cancer	Registries	≥ 3 cups/d vs. none/less than daily	1.32 (1.08, 1.62)	1, 2, 3, 4, 6, 7, 8, 9, 10, 11, 12, 13, 14, 15	9
Zhu et al., 2020	USA, China, Japan, Korea, and Singapore	58	M/F	1,117,156 (20,280)	8.6	Coffee	FFQ	Lung cancer	Registries or self-reports confirmed by medical record review	≥ 3 cups/d vs. none	1.36 (1.28, 1.44)	1, 2, 3, 5, 9, 16, 17, 18	8
Narita et al., 2018	Japan Public Health Center-based Prospective Study (JPHC Study)/Japan	40–69	M/F	87,079 (1668)	17	Coffee	FFQ	Lung cancer	Registries	≥ 5 cups/d vs. none	1.22 (0.90, 1.66)	1, 2, 3, 4, 13, 14, 17, 18, 19, 20, 21	8
Park et al., 2018	Multiethnic Cohort Study (MEC)/USA	59.5	M/F	167,720 (3954)	15.3	Coffee	FFQ	Lung cancer	Registries	≥ 4 cups/d vs. none	1.08 (0.92, 1.26)	1, 3, 9, 17, 20, 22, 23, 24, 25	9
Guertin et al., 2016	NIH-AARP Diet and Health Study/USA	62	M/F	457,366 (9196)	10.5	Coffee	FFQ	Lung cancer	Registries	≥ 6 cups/d vs. none	1.29 (1.15, 1.45)	1, 2, 3, 4, 6, 9, 13, 14, 16, 17, 20, 22, 23, 26, 27, 28, 29	8
Lukic et al., 2016	Norwegian Women and Cancer Study (NOWAC)/Norway	30–70	F	91,767 (819)	13.1	Coffee	FFQ	Lung cancer	Registries	> 7 cups/d vs. ≤ 1 cup/d	2.01 (1.47, 2.75)	3, 4, 5, 9, 20, 30, 31	9
Hashibe et al., 2015	Prostate, Lung, Colorectal, and Ovarian cancer screening trial (PLCO)/USA	55–74	M/F	96,024 (1137)	10	Coffee	DHQ	Lung cancer	Self-reported	≥ 2 cups/d vs. < 1 cup/d	1.10 (0.94, 1.28)	4, 32, 33, 34	8
Gnagnarella et al., 2013	Italian Continuous Observation of Smoking Subjects (COSMOS)/ Italy	≥ 50	M/F	4336 (178)	5.7	Coffee	FFQ	Lung cancer	Repeated annual screening computed tomography	≥ 5 cups/d vs. never	1.11 (0.47–2.56)	6, 35	6
Bae et al., 2013	Seoul Male Cancer Cohort Study (SMCC)/Korea	40–59	M	7009 (93)	16	Coffee	FFQ	Lung cancer	Registries	≥ 7 times/week vs. none	1.89 (0.94, 4.30)	–	6
Takezaki et al., 2003	Regional Cancer Registry in Aichi Prefecture (RCRAP)/Japan	40–79	M/F	5885 (51)	14	Coffee	FFQ	Lung cancer	Questionnaire	High vs. low	1.20 (0.60–2.40)	1, 2, 4, 36	6
Stensvold et al., 1994	Norway	35–54	M/F	42,973 (125)	10.1	Coffee	FFQ	Lung cancer	Registries	≥ 7 cups/d vs. ≤ 2 cups/d	1.90 (1.40, 2.70)	1, 37, 38	6
Jacobsen et al., 1986	Norway	NR	M/F	16,555 (177)	11.5	Coffee	Questionnaire	Trachea, bronchus, and lung cancer	Registries	≥ 7 cups/d vs. ≤ 2 cups/d	1.82 (1.27, 2.61)	–	6
Nomura et al., 1986	Japan	45–68	M	7355 (110)	10	Coffee	24-h dietary recall history	Lung cancer	Histologic examination	≥ 5 cups/d vs. none	1.55 (0.85, 2.81)	1, 4, 33, 37, 39	7

*CI* confidence interval, *DHQ* diet history questionnaire, *FFQ* food frequency questionnaire, *HR* hazard ratio, *NR* not reported, *OR* odds ratio, *RR* risk ratio.

Age = 1, sex = 2, body mass index = 3, smoking status = 4, number of pack-years smoked = 5, total energy intake = 6, dialect group = 7, interview year = 8, education level = 9, dietary beta-cryptoxanthin = 10, fried meat = 11, soy intake = 12, fruits intake = 13, vegetables intake = 14, second-hand smoke = 15, race/ethnicity = 16, alcohol consumption = 17, tea consumption = 18, PHC area = 19, physical activity = 20, isoflavone intake = 21, history of diabetes = 22, family history of cancer = 23, menopausal status = 24, menopausal hormone therapy = 25, health status = 26, supplement use = 27, marital status = 28, history of cardiovascular disease = 29, age at smoking initiation = 30, exposure to smoking in childhood = 31, smoking frequency = 32, smoking duration = 33, time since stopping smoking for past smokers = 34, baseline risk probability = 35, occupation = 36, cigarettes per day = 37, county of residence = 38, past smoking status = 39.

### Quality assessment

The quality of studies included in this systematic review and meta-analysis was examined by the Newcastle–Ottawa Scale (NOS). Based on this method, a maximum of nine scores can be awarded to each study. In our analysis, we considered scores of ≥ 6 as high-quality studies, otherwise, the study was deemed to have low quality. Table 1 indicates the results of the quality assessment of the eligible cohorts.

### Statistical analysis

All reported ORs, RRs, and HRs and their 95% CIs for risk of lung cancer were used to calculate the log RRs and their SEs. The overall effect size was calculated using the random-effects model, which incorporates between-study heterogeneity. Statistical between-studies heterogeneity was examined using Cochrane’s *Q* test and I-squared (I^2^). Publication bias was assessed by visual inspection of funnel plots. Formal statistical assessment of funnel plot asymmetry was carried out with Egger’s regression asymmetry tests. Sensitivity analysis was used to explore the extent to which inferences might depend on a particular study or group of studies. Statistical analyses were made with Stata MP, version 14. P-values < 0.05 were considered statistically significant.

To perform a dose–response analysis, we used studies that reported sufficient information. Studies were considered eligible if they reported the range or median/mean dose of coffee consumption (cups per day), the numbers of cases and participants/person-years, adjusted RRs and their 95% CIs across categories of coffee consumption. We divided the total number of cases, participants, and person-years by the number of categories if a study had not reported the sufficient information in each category. The linear dose–response association was measured using generalized least squares trend estimation, based upon the work of Greenland and colleagues^25,26^. The RR was calculated for a daily increase of one cup of coffee intake in each study. To pool the results of each study, a random-effects model was used. Restricted cubic splines with 3 knots according to Harrell’s recommended percentiles of distribution (10th, 50th, and 90th) were used to examine the potential nonlinear association^27^. The null hypothesis was tested by calculating a P-value for non-linearity of the meta-analysis. The test was conducted to check if the coefficient of the second spline was equal to 0.

## Results

**Figure 1:**
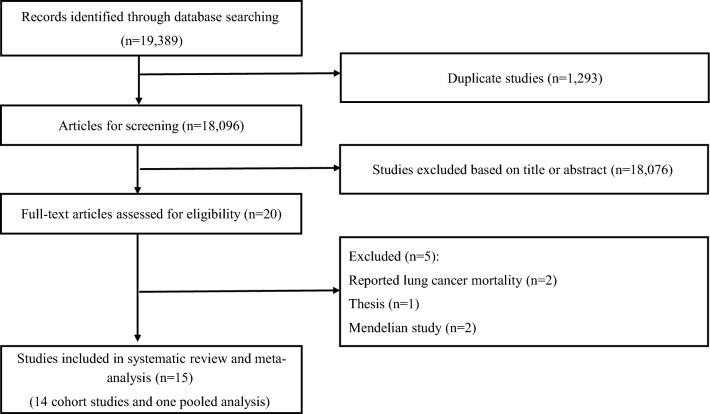
Flowchart of the study selection process.

Letters, reviews, meta-analyses, comments, animal studies, and ecological studies were excluded in the current systematic review and meta-analysis. Following our initial search, 19,389 articles were identified. After removing 1293 duplicates, 18,096 reports remained for further assessment. After title and abstract careful checking and review, 18,076 articles were excluded and 20 publications remained for full-text assessment. Five studies were excluded due to the following reasons: two studies had reported lung cancer mortality^23,24^. In addition, one thesis^28^ and two Mendelian studies^29,30^ were also excluded. Finally, a total of 14 prospective cohort studies^15–22,31–36^ and one pooled analysis^37^ were included in this systematic review and meta-analysis. Figure 1 illustrates the study selection process.

A recent pooled analysis by Zhu et al.^37^ included 17 cohort studies; however, 12 of them were unpublished data with no available full-texts. Therefore, we decided to analyze data once by including the study by Zhu et al.^37^ and excluding the 5 studies^17,20,21,31,32^ that overlapped with the Zhu et al., and once again by adding the 5 studies and excluding the study of Zhu et al. for better understanding of the association.

Narita et al.^17^ had reported effect sizes separately for men and women, however, we combined these two effect sizes and then, included in our analysis. Three studies had not reported the 95% CIs for the association between coffee consumption and risk of lung cancer^34–36^. Therefore, we derived relevant data for these studies from the previous meta-analysis^14^.

### Findings from the systematic review

#### Study characteristics

Overall, 14 cohort studies^15–22,31–36^ and one pooled analysis^37^ were included in the present systematic review (Table 1). These studies were reported from 1986 to 2020; four were from the United States^18,20,31,32^, three from Norway^22,34,36^, three from Japan^15,17,35^, one each from Thailand^19^, Italy^16^, Singapore^21^, and Korea^33^. The median follow-up duration ranged from 10 to 17.7 years. For the exposure assessment, 10 studies had used food frequency questionnaire^15–18,20–22,32–34^, 1 had collected data based on diet history questionnaire^31^ and one had used a structured questionnaire^19^. Others had reported using a questionnaire^36^ and 24-h dietary recall history^35^. For the outcome assessment, all, but four, of the included studies had used cancer registries. Outcome assessment in the PLCO study^31^ was self-reported and Nomura et al. had used histologic examination^35^. Based on the NOS, all included studies were of high quality.

### Findings from the meta-analysis

**Figure 2:**
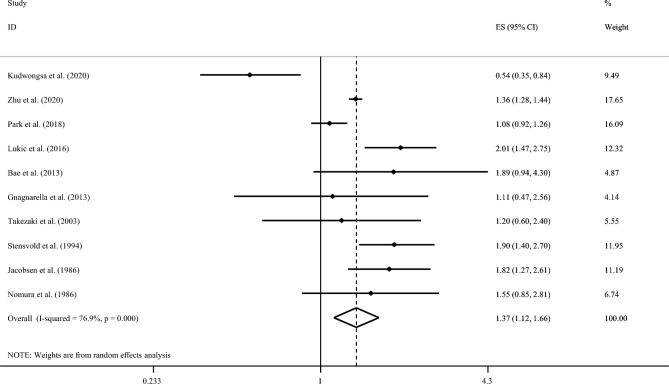
Forest plot of prospective cohort studies that examined the association between coffee consumption and risk of lung cancer using a highest vs. lowest analysis (including the study of Zhu et al.).

First, we examined the association by including data from 9 cohort studies not included in the pooled analysis paper of Zhu et al. along with the findings from the pooled analysis. The overall effect size based on these 10 studies^15,16,18,19,22,33–37^ revealed a statistically significant association between coffee consumption and risk of lung cancer (RR: 1.37; 95% CI: 1.12, 1.66; Fig. 2).

We also found an evidence of statistically significant between-study heterogeneity (I^2^ = 76.9%, P < 0.001). No evidence of publication bias was seen (P = 0.93).

**Figure 3:**
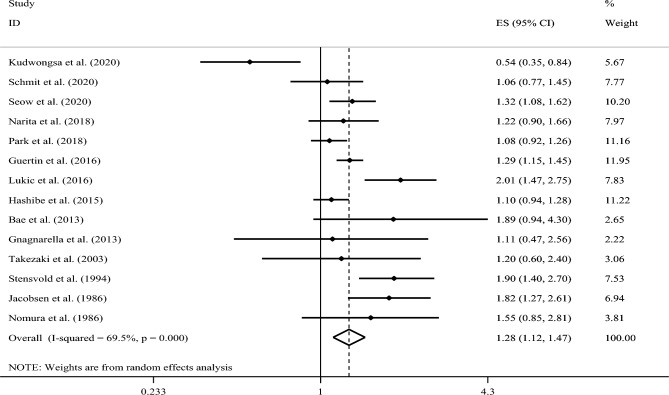
Forest plot of prospective cohort studies that examined the association between coffee consumption and risk of lung cancer using a highest vs. lowest analysis (excluding the study of Zhu et al.).

In a further analysis, we excluded the study of Zhu et al. and included 14 cohort studies in the analysis. Combining 14 effect sizes from 14 studies^15–22,31–36^, we observed a statistically significant positive association between coffee consumption and risk of lung cancer (RR: 1.28; 95% CI: 1.12, 1.47; Fig. 3).

However, a significant between-study heterogeneity was found (I^2^ = 69.5%, P < 0.001). A sensitivity analysis showed that no particular study had a significant influence on the summary effects. In addition, we observed no proof of significant publication bias using Egger’s test (P = 0.58). (Funnel plot has provided as Supplementary Fig. 1).

To find sources of heterogeneity, we performed subgroup analyses based on fixed-effects model. In the subgroup analyses, we found that sex, follow-up duration, and country might explain between-study heterogeneity (Table 2).

**Table 2:** Subgroup analyses for the association between coffee consumption and risk of lung cancer.

Variables	Effect sizes, n	I^2^, %	*Q* test	RR (95% CI)	*P*_between_
Sex					0.004
Men	2	0	0.68	1.67 (1.05, 2.68)	
Women	1	–	–	2.01 (1.47, 2.75)	
Both	11	68.1	0.001	1.21 (1.13, 1.29)	
Follow-up duration (year)					0.02
< 15	8	66.2	0.001	1.33 (1.22, 1.44)	
≥ 15	5	74.2	0.004	1.13 (1.01, 1.26)	
Country					0.008
USA	4	36.2	0.19	1.17 (1.09, 1.27)	
Non-USA	10	70.9	< 0.001	1.42 (1.26, 1.59)	
Exposure assessment					0.68
FFQ	11	58.1	0.008	1.25 (1.17, 1.33)	
Non-FFQ	3	89.3	< 0.001	1.18 (0.92, 1.52)	
Outcome assessment					0.13
Registries	10	77	< 0.001	1.27 (1.19, 1.37)	
Non-registries	4	69.5	< 0.001	1.13 (0.98, 1.30)	
Adjust for smoking status					0.45
Yes	8	48.3	0.06	1.26 (1.17, 1.36)	
No	6	82.5	< 0.001	1.19 (1.06, 1.35)	

*CI* confidence interval, *RR* rate or risk ratio, *FFQ* food frequency questionnaire.

A significant positive association between coffee consumption and risk of lung cancer was seen in women (RR: 2.01; 95% CI: 1.47, 2.75), men (RR: 1.67; 95% CI: 1.05, 2.68), and both sexes (RR: 1.21; 95% CI: 1.13, 1.29). In addition, we observed a significant positive association between coffee consumption and risk of lung cancer in studies with < 15-year duration of follow-up (RR: 1.33; 95% CI: 1.22, 1.44), as well as those with ≥ 15-year of follow-up (RR: 1.13; 95% CI: 1.01, 1.26), those conducted in USA (RR: 1.17; 95% CI: 1.09, 1.27), and those conducted in non-USA countries (RR: 1.42; 95% CI: 1.26, 1.59). In addition, there was a significant positive association between coffee consumption and risk of lung cancer among studies that adjusted analysis for smoking status (RR: 1.26; 95% CI: 1.17, 1.36) and those that did not adjust for smoking status (RR: 1.19; 95% CI: 1.06, 1.35).

**Figure 4:**
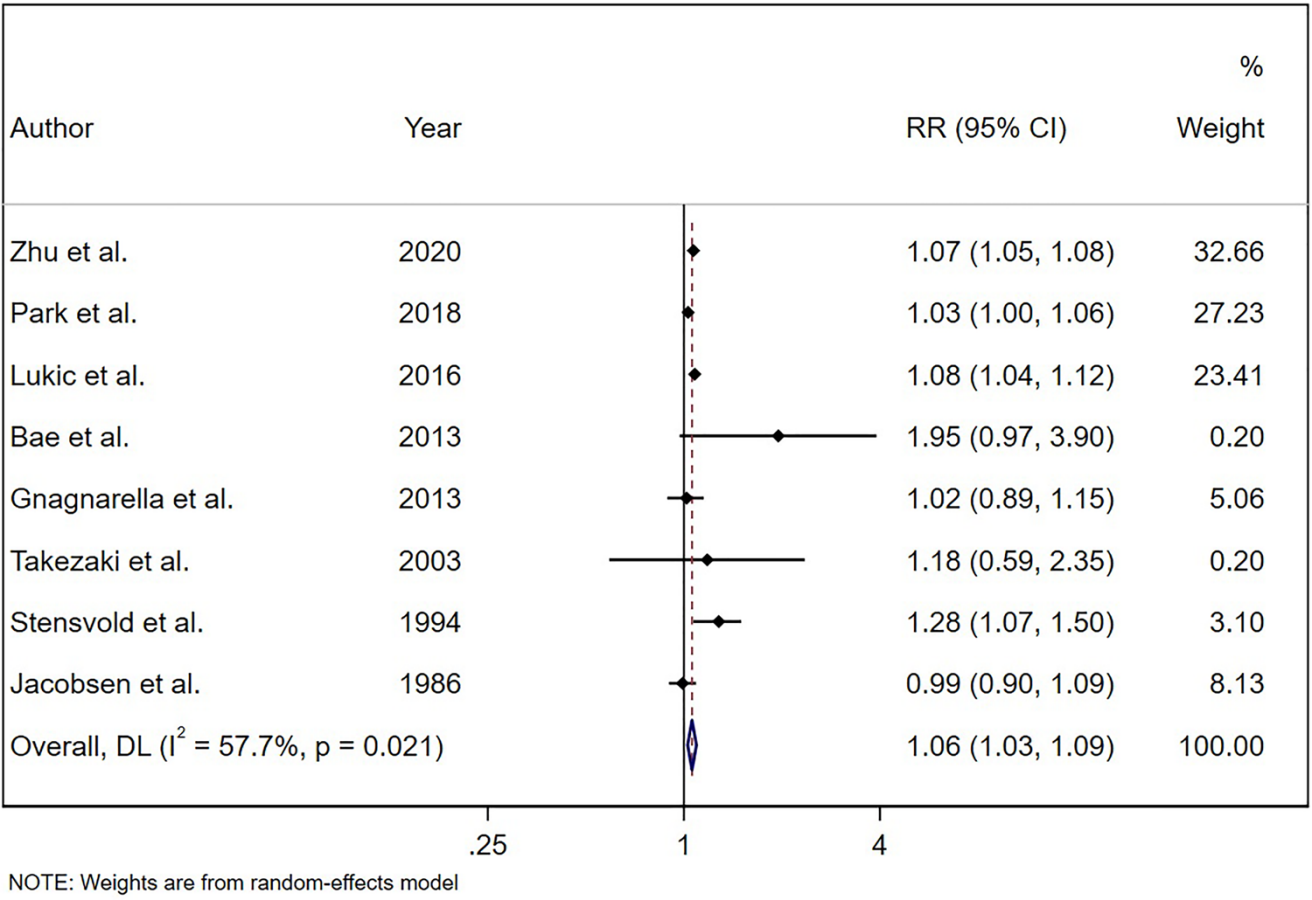
Relative risk of lung cancer for a one cup/day increment in coffee consumption based on 8 studies.

A total of 3 studies were excluded from dose–response analysis as they did not provide sufficient information even after receiving two email requests^19,20,35^. Therefore, 11 studies remained for further analyses. Results from 8 studies including Zhu et al. study demonstrated that each one cup/day increase in coffee consumption was associated with a 6% higher risk of lung cancer (RR: 1.06; 95% CI: 1.03, 1.09; Fig. 4).

**Figure 5:**
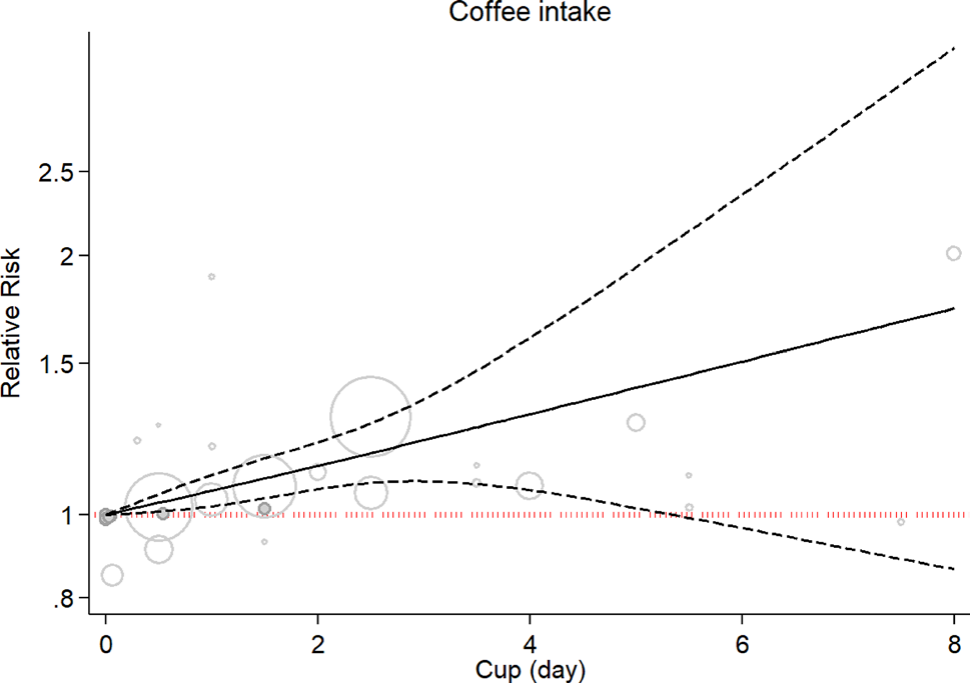
Nonlinear dose–response association between coffee consumption and the risk of lung cancer (P non-linearity = 0.82) based on 8 studies.

The risk of lung cancer increased linearly with coffee consumption of approximately 1–5 cups per day in a nonlinear dose–response analysis (P nonlinearity: 0.94; P dose–response: 0.001; Fig. 5).

**Figure 6:**
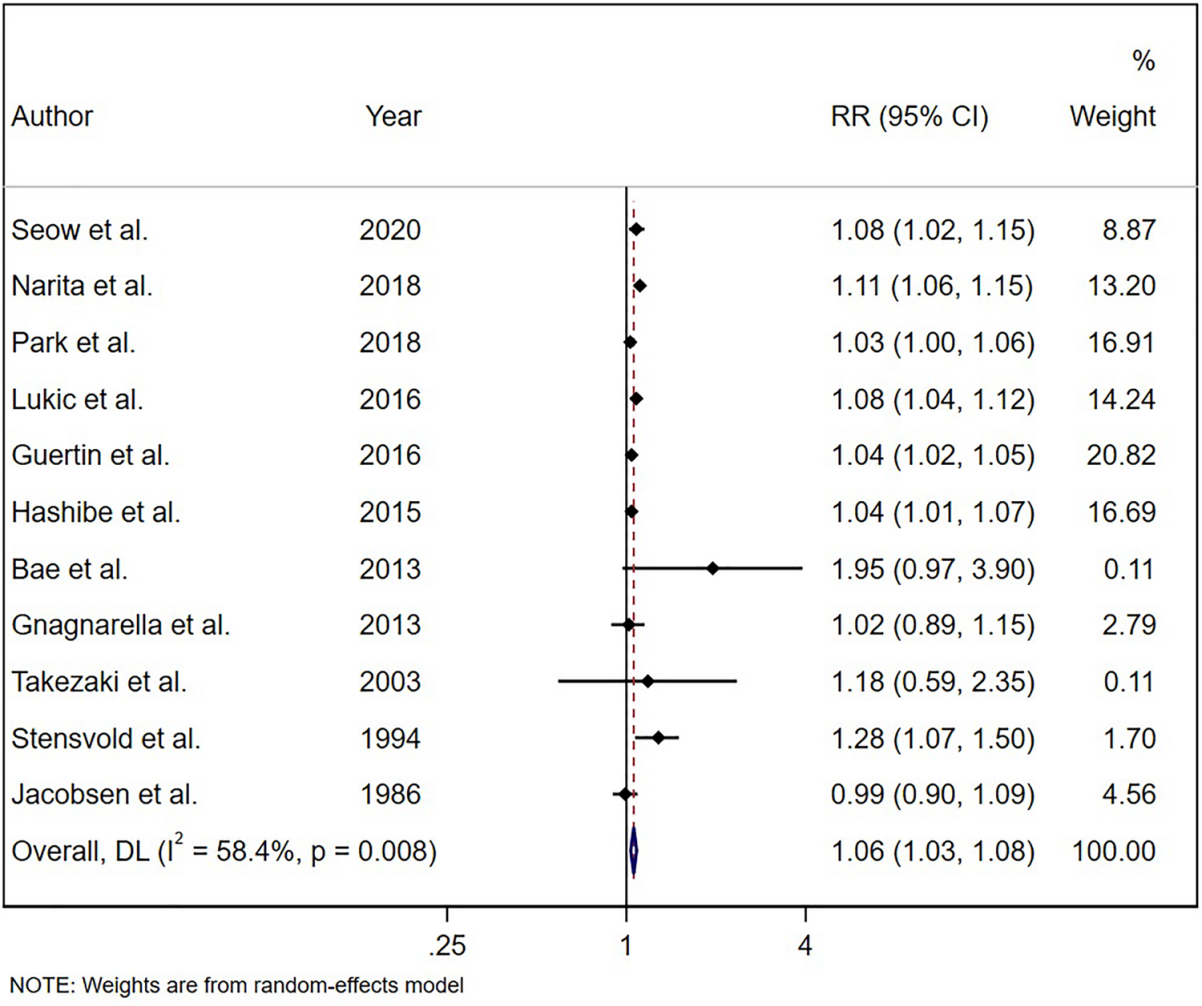
Relative risk of lung cancer for a one cup/day increment in coffee consumption based on 11 studies.

Such associations were observed when we excluded the study of Zhu et al. and included 11 cohort studies in the linear dose–response analysis (RR: 1.06; 95% CI: 1.03, 1.08, P_nonlinearity_ = 0.65; Fig. 6).

**Figure 7:**
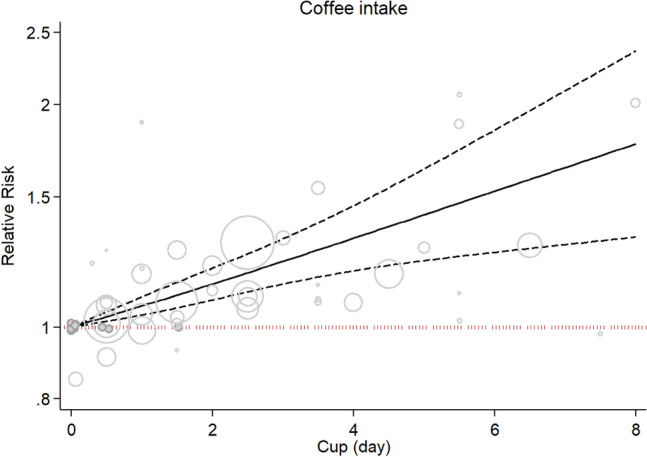
Nonlinear dose–response association between coffee consumption and risk of lung cancer based on 11 studies.

There was also a linear association between coffee consumption and risk of lung cancer (P nonlinearity = 0.94, P dose–response: 0.001; Fig. 7).

## Discussion

This systematic review and meta-analysis on 14 prospective cohort studies and a pooled analysis indicated a significant positive association between coffee consumption and risk of lung cancer. It was also found that an increase of one cup of coffee per day was linked to a higher risk of lung cancer, according to the dose–response analysis. To the best of our knowledge, this is the most comprehensive and updated meta-analysis about coffee consumption and risk of lung cancer.

Lung cancer imposes a great burden on the health care system. Although smoking is a well-established risk factor for this condition, dietary factors also play an important role. Fruit and vegetables consumption was inversely associated with risk of lung cancer in earlier studies^38^. In addition, consumption of American/Western dietary pattern has been associated with 45% elevated risk of lung cancer^39^. Coffee is a regular drink in most parts of the world and evaluating its contribution to human health is of high importance. We found that coffee consumption was associated with a greater risk of lung cancer. Such findings were also reported from a meta-analysis on 5 cohort studies in 2010^13^ and a meta-analysis on 8 cohort studies in 2016^14^. Another previous meta-analysis conducted in 2016 also indicated a significant positive association between coffee consumption and risk of lung cancer^11^. Data from a prospective cohort study on Women’s Health Initiative (WHI) observational study reported a significant elevated risk of lung cancer for regular, decaffeinated, and total coffee consumption^28^. In contrast to our findings, a meta-analysis on 8 case–control studies revealed no significant association between coffee consumption and risk of lung cancer^13^. In addition, a case–control study reported a significant inverse association between weekly compared to never coffee consumption and risk of lung cancer^40^. In the meta-analysis published in 2016, when the authors combined prospective cohort and case–control studies, without controlling for smoking, a significant association was seen between coffee consumption and risk of lung cancer; however, after restricting the analysis to studies that adjusted for smoking, no significant association was observed^14^. The discrepant findings can be explained by the difference in the number of studies included in different meta-analyses. In addition, combining effect sizes from case–control studies with those from prospective cohort studies would result in misleading findings. We included a total of 14 cohort studies in the current analysis with a total population of 1,061,854 people and 19,643 incident cases of lung cancer^15–22,31–36^. Comparing these figures with the numbers in previous meta-analyses, it is clear that we had a larger number of people and incident cases in the current analysis, which make our findings more valid and reliable.

The mechanisms through which coffee consumption might affect the risk of lung cancer still remain to be identified. Some biochemically active components of coffee might influence cancer risk. Coffee can be an important dietary source of acrylamide which is a genotoxic agent. Roasting process helps increasing acrylamide content of coffee^41^. Acrylamide can cause DNA damage in mammalian tissues and induce oxidative stress and thus trigger cancer cell formation^42^. Caffeine is a widely known substance in coffee which might have mutagenic effect on cancer development^43^. However, some studies have also reported anti-cancer properties for caffeine^44^. Despite these contents of coffee, it might also have cancer-protective effects. Cafestol and Kahweol may potentially inhibit tumor growth by blocking or diminishing neoangiogenesis, however, they also increase cardiovascular risk by raising the concentration of serum lipids^45^. Overall, it seems that coffee with its ingredients might be beneficial or detrimental to different cancers and further studies are needed to elucidate the relevant mechanisms.

Our study has several strengths. Restricting the analysis to prospective cohort studies as well as large number of included studies and participants compared to previous ones are among several strengths. In addition, findings from a recent pooled analysis were also used carefully without overlapping the included studies. This has been resulted to include a large number of individuals in the analysis, in particular from cohort studies for which there are no original report about coffee consumption and lung cancer. However, some limitations must be noted when interpreting our results. This systematic review and meta-analysis was performed based on observational studies with their inherent limitations. Therefore, it is difficult to make a conclusive decision about the causal association between coffee consumption and risk of lung cancer. In addition, for most included studies, coffee consumption was assessed using a food frequency questionnaire. Therefore, measurement error and misclassification of study participants in terms of exposure were unavoidable. Both non-differential misclassification and measurement errors attenuate the relative risk. Furthermore, the present systematic review and meta-analysis included studies that had enrolled subjects from different countries with different dietary habits and racial factors, which could be associated with different risks of lung cancer. Despite adjustment for several potential confounders in primary studies, the possibility of residual confounding cannot be ignored. The quality of the included studies and generalizability of the results should also be noted. Finally, we were unable to examine the association between different types of coffee and risk of lung cancer, because included studies had not reported such information separately.

In conclusion, this systematic review and meta-analysis indicated a significant positive association between coffee consumption and risk of lung cancer. Further studies, especially with prospective design, are required to expand our knowledge on the association between coffee consumption and risk of lung cancer.

### Supplementary Information

Supplementary Figure 1.

## Data Availability

The dataset used and analyzed during the current study is available from the corresponding author on a reasonable request.

## Abbreviations

CIs: Confidence intervals
HRs: Hazard ratios
NOS: Newcastle–Ottawa Scale
ORs: Odds ratios
RRs: Rate or risk ratios
WHI: Women’s Health Initiative
