# Supplementary material for: A systematic review and dose–response meta-analysis of prospective cohort studies on coffee consumption and risk of lung cancer
Source: Sci Rep. 2024 Jul 1;14:14991. doi: 10.1038/s41598-024-62619-6 (PMC11217372; doi:10.1038/s41598-024-62619-6)
Supplement: Supplementary file 1 — Supplementary Figure 1. [file 41598_2024_62619_MOESM1_ESM.pptx]

## Slide 1
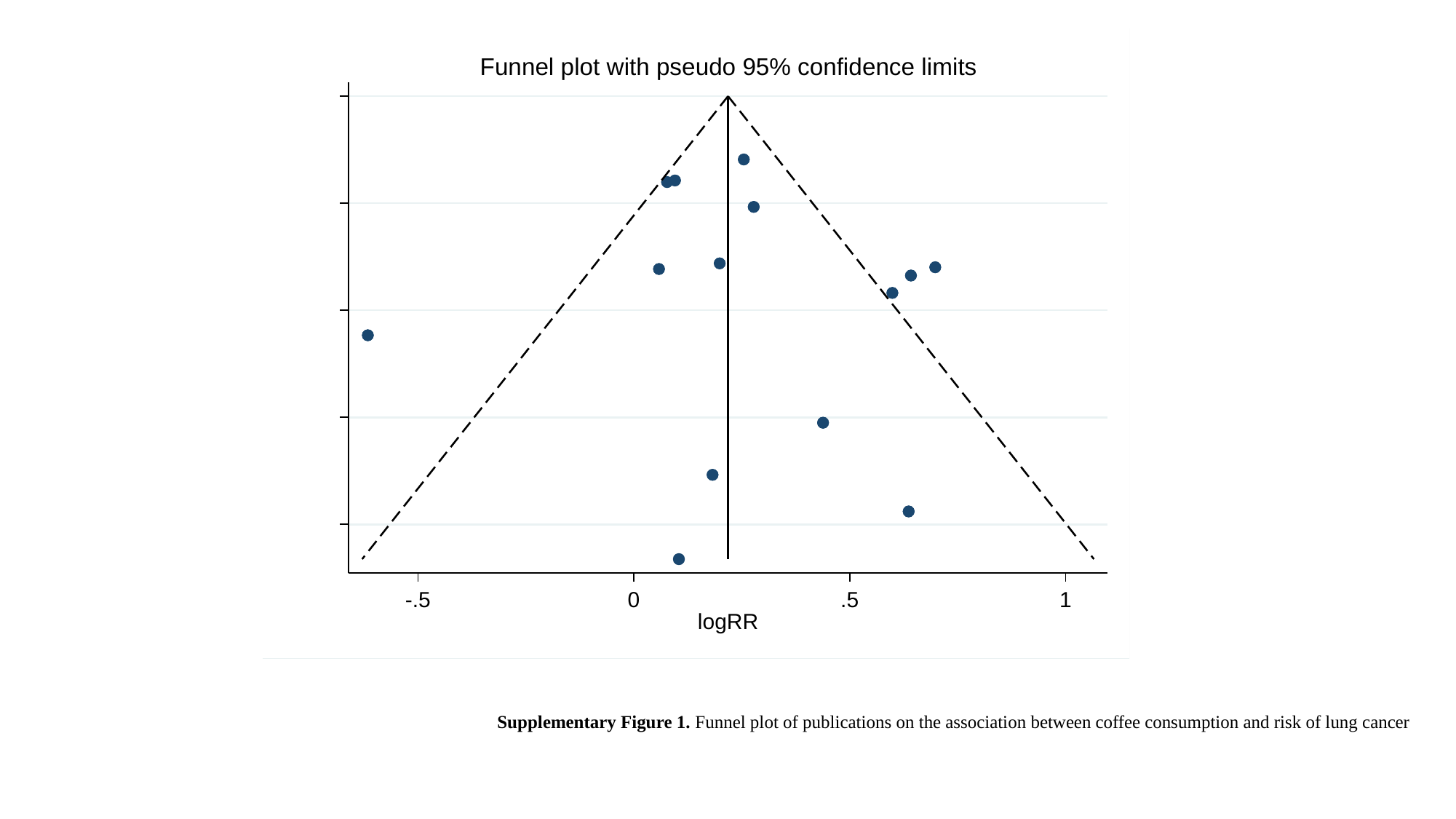

Supplementary Figure 1. Funnel plot of publications on the association between coffee consumption and risk of lung cancer
